# Modification of titanium orthopedic implants with bioactive glass: a systematic review of *in vivo* and *in vitro* studies

**DOI:** 10.3389/fbioe.2023.1269223

**Published:** 2023-11-15

**Authors:** Jin Liang, XinYue Lu, XinRu Zheng, Yu Ru Li, XiaoYu Geng, KeXin Sun, HongXin Cai, Qi Jia, Heng Bo Jiang, Kai Liu

**Affiliations:** ^1^ Department of Oral and Maxillofacial Surgery, School of Stomatology, Shandong First Medical University, Jinan, Shandong, China; ^2^ The CONVERSATIONALIST Club and Department of Stomatological Technology, School of Stomatology, Shandong First Medical University, Jinan, Shandong, China; ^3^ Department and Research Institute of Dental Biomaterials and Bioengineering, Yonsei University College of Dentistry, Seoul, Republic of Korea; ^4^ School of Basic Medicine, Shandong First Medical University, Jinan, Shandong, China

**Keywords:** bone regeneration, surface modification, biomaterials, tissue engineering, bioglass, bioactive materials

## Abstract

Bioactive glasses (BGs) are ideal biomaterials in the field of bio-restoration due to their excellent biocompatibility. Titanium alloys are widely used as a bone graft substitute material because of their excellent corrosion resistance and mechanical properties; however, their biological inertness makes them prone to clinical failure. Surface modification of titanium alloys with bioactive glass can effectively combine the superior mechanical properties of the substrate with the biological properties of the coating material. In this review, the relevant articles published from 2013 to the present were searched in four databases, namely, Web of Science, PubMed, Embase, and Scopus, and after screening, 49 studies were included. We systematically reviewed the basic information and the study types of the included studies, which comprise *in vitro* experiments, animal tests, and clinical trials. In addition, we summarized the applied coating technologies, which include pulsed laser deposition (PLD), electrophoretic deposition, dip coating, and magnetron sputtering deposition. The superior biocompatibility of the materials in terms of cytotoxicity, cell activity, hemocompatibility, anti-inflammatory properties, bioactivity, and their good bioactivity in terms of osseointegration, osteogenesis, angiogenesis, and soft tissue adhesion are discussed. We also analyzed the advantages of the existing materials and the prospects for further research. Even though the current research status is not extensive enough, it is still believed that BG-coated Ti implants have great clinical application prospects.

## 1 Introduction

Bioactive glass (BG) can repair, restore, replace, and help regenerate tissue through the combination of tissues and materials. BG has uniform particle size and great adhesion with irregular sizes and shapes. Furthermore, its inherent biocompatibility and high strength make it an ideal biomaterial (Manam et al., 2017). After contact with biological fluids, some ions are released on the surface of the BG particles, which can regulate the osmotic pressure and pH value around the implant, thereby damaging the cell wall structure of bacteria and inducing antibacterial activity (Allan et al., 2002; Coraça-Huber et al., 2014; Drago et al., 2018). BGs also have appropriate biodegradability and particles are easily absorbed. In addition, this material has good bioactivity, which can promote osteoinduction and thus rapidly form bone-like structures. However, when compared to human bone tissue, BG is more brittle. Due to its poor mechanical properties, BG is not suitable for the load-bearing areas (Cannillo et al., 2009; Yanovska et al., 2011). The mentioned properties make it ideal for use in toothpaste, bone grafts, scaffolds, drug delivery systems, soft tissue engineering, and biomaterial coatings (Hench, 2006; Asif et al., 2014).

Titanium (Ti) alloys have better biocompatibility than other metal implants. Ti does not cause rejection after direct contact with bone tissue nor does it have negative effects or other bioelectronic reactions on biological organs. When compared with stainless steel and cobalt-based metals, Ti has lower modulus and better corrosion resistance while the corrosion resistance of metals and the biocompatibility of corrosion products can reflect metal’s own biocompatibility (Long and Rack, 1998). Their inertness made them virtually unreactive to the surrounding tissue environment, resulting in low cytotoxicity. The hydroxyapatite (HA) layer is often coated on the Ti alloy surface, which leads to the combination with the host collagen fiber, demonstrating Ti alloy’s osteointegration. The oxide layer on their surface is equipped with excellent corrosion resistance (Escalas et al., 1976). For mechanical property aspects, Young’s modulus of Ti alloy is much smaller than that of other metallic biomaterials, such as stainless steel, thereby reducing the stress shielding effect (Niinomi, 1998). Due to their excellent biocompatibility, corrosion resistance, and mechanical properties, Ti alloys can be applied as orthopedic and stomatological implants for arthroplasty and implantology. However, due to their intrinsic inertness, Ti alloys cannot form a close connection at the interface between the implant and host tissue. Meanwhile, the low wear resistance causes implants to loosen (Long and Rack, 1998). The inability of Ti alloys to achieve both shear strength and ductility greatly limits their application as implant materials in joint replacement (Chen and Thouas, 2015). The physical and chemical properties of the implant surface are crucial and play an important role in the osseointegration process between the bone and the implant. Therefore, surface modification of titanium implants can greatly reduce their limitations in clinical application (Geng et al., 2021a).

The surface modification of Ti using BG can improve osteointegration and osteogenesis (Gomez-Vega et al., 2001; López et al., 2016), which combines the substrates’ excellent mechanical properties with BG coatings’ biological properties (Solai et al., 2011). In fact, HA is still a common material for titanium alloy coatings. Apatite has the same inorganic composition as bone tissue and has been widely used in the field of bone transplantation and studied as a coating material (Geng et al., 2021b). Both BG and HA have good biological properties and excellent osteoconductivity. However, BG has better osteogenesis properties than HA coating materials (Dhinasekaran et al., 2021). BG coatings lead to apatite layer formation on the surface and thus improve close integration with both human hard and soft tissues (Sanz-Herrera and Boccaccini, 2011) and help in bone growth (Moreira et al., 2018; Vuornos et al., 2019). Furthermore, the ions released by BGs in bodily fluids can stimulate angiogenesis and wound healing (Cohrs et al., 2019; Zhang et al., 2019). Multiple manufacturing technologies have been investigated to coat Ti alloys with BG, such as electrophoretic deposition (Estrada-Cabrera et al., 2019), electrochemical deposition (Balamurugan et al., 2009), pulsed laser deposition (PLD) (Wang et al., 2018), dip coating deposition (Safaee et al., 2021), magnetron sputtering (Berbecaru et al., 2010), thermal spraying (Herman et al., 2000), and laser cladding (Comesana et al., 2010). Doping different ions in BG can improve the specific properties of the coating, such as magnesium ions (Mg^2+^), zinc ions (Zn^2+^), and strontium ions (Sr^2+^)*.* Many recent studies have shown that Sr^2+^ can promote bone formation and inhibit osteoclast absorption; therefore, this element is often doped into implants or their coatings to improve the osteogenic performance of the implants (Geng et al., 2021a; Geng et al., 2022). Besides, BG coatings, when combined with other biomaterials, can improve biological properties. Drug-loaded chitosan BG coatings exhibit good cellular activity, antimicrobial capacity, and osteogenic activity (Patel et al., 2012).

In a previously published review relevant to BG coatings, Oliver et al. (2019) summarized the performance improvement of BG coatings on medical metallic implants. Maximov et al. (2021) listed different methods of BG preparation as well as coating technologies. Baino and Verné (2017) specifically discussed the different clinical areas of application of BG coatings on biomedical implants. In this systematic review, the authors have searched and screened relevant articles and summarized and analyzed the characteristics of the included studies, the manufacturing technologies of BG coatings on Ti implants, and the properties of BG coatings. Previous research status that included study types is also included in this review. This systematic review aims to evaluate the properties of BG-coated Ti implants *versus* bare Ti implants and systematically adds up previous coating technology and relevant parameters as the influencing factors for BG coatings on Ti implants, providing a theoretical basis for future studies.

## 2 Materials and methods

### 2.1 Inclusion and exclusion criteria

**FIGURE 1:**
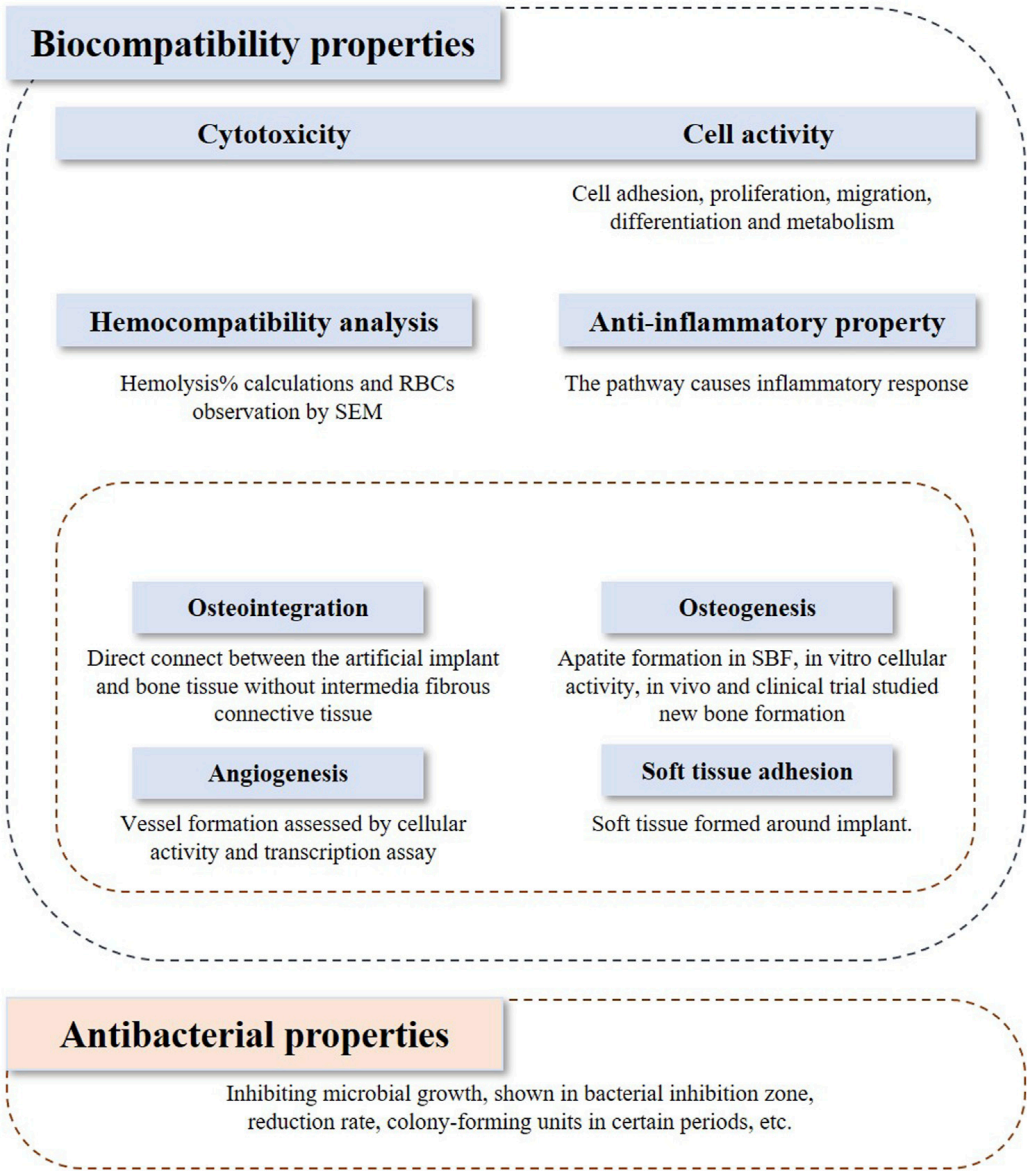
Outcome indicators for assessment.

The inclusion and exclusion criteria were framed based on the PICOS model. *In vivo* studies and clinical trials, and *in vitro* studies that investigated both biocompatibility and bioactivity were included in the assessment. Studies applying Ti or its alloys coated with bioactive glasses or the composite coatings on the Ti implant containing bioactive glasses were included in this review. The outcome indicators discussed in this review contain biocompatibility, bioactivity, and antibacterial properties, which are shown in Figure 1. Biocompatibility refers to 1) cytotoxicity and cell activity, 2) hemocompatibility, 3) anti-inflammatory properties, and 4) bioactivity. Bioactivity comprises 1) osteointegration, 2) osteogenesis, 3) angiogenesis, and 4) soft tissue adhesion.

Articles published in the last 10 years were included. There are no restrictions on the research type. Articles not published in English and whose full texts were unavailable were excluded.

### 2.2 Literature search and screening

Four databases, namely, PubMed, Embase, Scopus, and Web of Science, were searched in this study. The search strategy is shown in Table 1.

**TABLE 1:** Searching strategies and results in the database.

Database	Searching strategy^a^	Result
**PubMed**	(((bioglass [Title/Abstract]) OR (bioactive glass [Title/Abstract]))AND ((ti [Title/Abstract]) OR (titanium [Title/Abstract])))	119
**Embase**	(titanium:ti,ab, kw OR ti:ti,ab,kw) AND (bioglass:ti,ab, kw OR bioactive glass:ti,ab,kw)	119
**Web of Science**	(TS=(titanium) OR TS=(ti)) AND (TS=(bioglass) OR TS=(bioactive glass))	566
**Scopus**	(TITLE-ABS-KEY (titanium) OR TITLE-ABS-KEY (ti)) AND (TITLE-ABS-KEY (bioglass) OR TITLE-ABS-KEY(Bioactive glass))	493
**After Duplicates were removed**		816

^a^ Time filters were set from 2013 to present.

That the bold values indicates the number of studies retrieved in different databases according to the searching strategy.

Duplicates were removed using Endnote X9.3.2. The first screening was performed by filtering the title and abstract and inclusion of studies was determined after reading the full texts. The screening was done following the inclusion and exclusion criteria and was performed by two independent authors. Any conflict was resolved by a third author.

### 2.3 Data extraction

Data were collected using Microsoft Excel. The extracted data included substance material and samples’ shapes and sizes, glass models and composites, experimental subjects, manufacturing methods, and special process parameters. Other included information can be seen in detail in the following contents.

The data were extracted independently by two researchers, and any problems were solved through discussion and a third author's help.

## 3 Characteristics of included studies

### 3.1 Basic information

**FIGURE 2:**
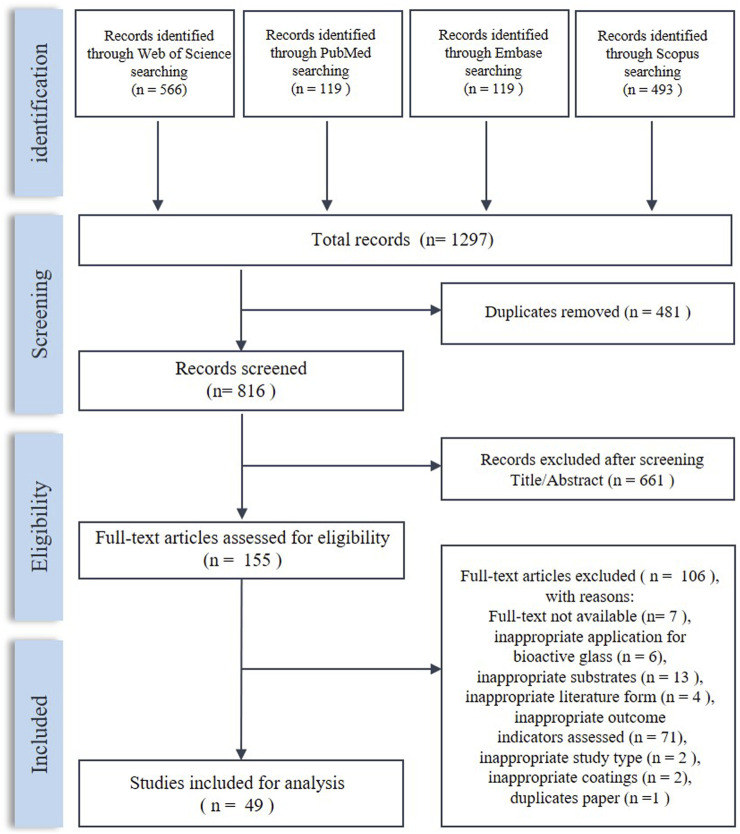
Flowchart for literature searching.

The process of literature screening is shown in Figure 2. After screening, 49 articles were included in this systematic review. The characteristics of the included studies are shown in Table 2.

**TABLE 2:** Characteristics of included studies.

Study ID			Titanium alloy substrate		Sample shape and size		Glass model	Glass component (wt%)								Other components		Manufacturing method		Special process		Ref.	
								SiO_2_		Na_2_O		CaO		P_2_O_5_									
Shaikh2019			Ti-6Al-4V		—		Bioglass 45S5	45		24.5		24.5		6		—		Melt-quenching		—		Shaikh et al. (2019)	
Catauro2016			Pure Ti (commercially grade 4)		Disks; diameter: 8 mm, thickness: 2.20 mm		—	—		—		—		—		—		Sol–gel		—		Catauro et al. (2016)	
Ananth2013			Ti-6Al-4V		Plate: 10 × 10 × 2 mm^3^		—	54		—		24		14		MgO: 8		—		Samples pre-deposited zirconia coating and composited with zirconia in different ratios		Ananth et al. (2013)	
Dhinasekaran2021			Pure Ti		Plate: 10 × 10 × 0.25 mm^3^		Bioglass 45S5	44.98		24.53		24.49		6		—		Sol–gel		—		Dhinasekaran et al. (2021)	
Fu2017 (a)			—		Plate: 10 × 10 × 1.2 mm^3^		—	—		—		—		—		—		Sol–gel		Doped with Ag		Fu et al. (2017)	
Gaweda2018			—		—		Black glasses	—		—		—		—		—		Sol–gel		—		Gaweda et al. (2018)	
Patel2019			Pure Ti		Plate: 10 × 10 × 1 mm^3^		—	—		—		—		—		—		—		Composited with chitosan		Patel et al. (2019)	
Su2019			Ti-6Al-4V		—		—	100		—		—		—		—		Sinter–granulation		Composited with HA		Su et al. (2019)	
								50		—		50		—		—							
								50		—		30		20		—							
								50		10		30		10		—							
Fu2017 (b)			Titanium (grade TA2)		Plate: 10 × 10 × 1.2 mm^3^		—	—		—		—		—		—		Sol–gel		Doped with strontium		Fu et al. (2017)	
Ledda2016			Pure Ti		Plate: 1 × 1 cm^2^		RKKP glass-ceramic	43.68		4.53		31.3		11.1		MgO: 2.78, K_2_O: 0.19, CaF_2_: 4.92, La_2_O_3_: 0.5, and Ta_2_O_5_: 1		Sol–gel		—		Ledda et al. (2016)	
Chen, X. C.2014			Ti-6Al-4V		Plate: 10 × 12 × 2 mm^3^; cylinders; diameter: 25.4 mm, length: 25.4 mm		CaO–MgO–SiO_2_-based multiphase glass-ceramic M2	49.13		—		43.19		—		MgO: 7.68		Sol–gel		—		Chen et al. (2014)	
Ordikhani, F.2014			Titanium (biomedical grade)		Plate: 10 × 20 × 0.45 mm^3^		—	45		24.5		24.5		6		—		Melt-drive		Composited with chitosan and vancomycin		Ordikhani and Simchi (2014)	
Palangadan, R.2014			Ti-6Al-4V		Plate: 15 × 10 × 2 mm^3^		Bioactive triphasic glass-ceramic composition (HASi)	34.2				44.9		16.3		MgO: 4.6, CaF_2_: 0.5		Sol–gel		—		Palangadan et al. (2014)	
Ordikhani2016			Titanium		Plate: 10 × 20 × 0.45 mm^3^		—	45		24.5		24.5		6		—		Melt-drive		Multilayer nanocomposite coating of bioactive glass with chitosan and vancomycin		Ordikhani et al. (2016)	
Popa2017			Pure Ti		Plate: 10 × 10 mm^2^		—	37.8		—		33.1		13		MgO: 10, CaF_2_: 0.8, and ZnO: 5.3		Melt-drive		—		Popa et al. (2017)	
Rastegari2019			Ti-6Al-4V		—		SiO_2_–CaO–MgO bioactive glasses	—		—		—		—		—		Coprecipitation		Composited with chitosan		Rastegari and Salahinejad (2019)	
Boschetto2020			Ti-6Al-4V (grade 5)		—		Bioglass 45S5	—		—		—		—		—		—		Composited with chitosan and polyethylene oxide nanofibers		Boschetto et al. (2020)	
Lung2021			Pure Ti (grade 2)		Disks; diameter: 25.4 mm; and thickness: 5.1 mm		58S	55.6		—		33.1		11.3		—		Sol–gel		Doped with silver, cobalt oxide, and titanium dioxide		Lung et al. (2021)	
							Ag56S	52.8		—		32.6		11.2		Ag: 3.4							
							Co56S	53.4		—		32.9		11.3		CoO: 2.4							
							Ti56S	53.3		—		32.9		11.3		TiO_2_: 2.5							
							All52S	48.5		—		32.2		11.1		TiO_2_: 2.5, CoO: 2.3, Ag: 3.4							
Matter2021			Ti-6Al-4V (medical grade)		Disks; diameter: 15 mm, thickness: 1 mm		—	—		—		—		—		—		Flame-made		Composited with cerium oxide; doped with strontium oxide and Zn and then composited with cerium oxide		Matter et al. (2021)	
Rau2020			Pure Ti		Plate: 1 × 1 cm^2^		Silicate glasses 13–93	56.6		5.5		18.5		3.7		MgO: 4.6 and K_2_O: 11.1		Melt-quenching		—		Rau et al. (2020)	
							Borate glasses 13–93-B3	—		5.5		18.5		3.7		MgO: 4.6, K_2_O: 11.1, and B_2_O_3_: 56.6							
Abushahba2020			Pure Ti (grade 5)		Plate: 10 × 10 × 1 mm^3^		Bioglass 45S5	45		24.5		24.5		6		—		—		Doped with zinc oxide		Abushahba et al. (2020)	
							Zn4	42.4		24.1		22.4		5.9		ZnO: 5.2							
Nesabi2021			Pure Ti (medical grade)		Disks; diameter: 12 mm, thickness: 1 mm		58S	58		—		38		4		—		Sol–gel		Samples only or samples and bioglass or both were alkali treated with sodium hydroxide		Nesabi et al. (2021)	
Ye2017			Ti-6Al-4V (medical grade 23)		Cylinders; diameter: 10 mm, height: 10 mm		—	—		—		—		—		—		Sol–gel		Samples pre-deposited a silica interlayer		Ye et al. (2017)	
Safaee2021			Pure Ti (commercially medical grade)		Plate: 10 × 10 × 1 mm^3^		58S	58		—		33		9		—		Sol–gel		—		Safaee et al. (2021)	
Zhang2016			Ti-6Al-4V		Plate: 10 × 10 × 2 mm^3^		—	75.6		—		13.2		11.2		—		Sol–gel		Samples pre-deposited ZrO_2_ coatings		Zhang et al. (2016)	
Avcu2018			Ti-6Al-4V		Plate: 76.2 × 25.4 × 2 mm^3^		Vitryxx^®^ Bioactive Glass (bioactive glass Bioglass 45S5^®^)	45 ± 5		24.5 ± 3		24.5 ± 3		6 ± 2		—		—		Composited with chitosan		Avcu et al. (2018)	
							Nano-bioglass (Schott NF-180 glass)	55		—		—		—		B_2_O_3_: 10, Al_2_O_3_: 10, BaO: 25							
Guimarães2020			Pure Ti (grade 4)		Plate: 4 × 2 mm^2^		—	—		—		—		—		—		Sol–gel		Deposited potassium hydroxide coatings based on bioglass coatings		Guimarães et al. (2020)	
Zarghami2020			Pure Ti (biomedical grade)		Plate: 5 × 5 × 0.7 mm^3^		Bioactive glass nanoparticles (BGNs)	45				49		6		—		Wet synthesized		Composited with chitosan–vancomycin		Zarghami et al. (2020)	
Costa2020			Pure Ti (grade 2)		Disks; diameter: 10 mm, thickness: 2 mm		—	—		—		—		—		—		—		—		Costa et al. (2020)	
Zarghami2021			Pure Ti (biomedical grade)		Plate: 5 × 5 × 0.7 mm^3^		—	—		—		—		—		—		—		Composited with chitosan, vancomycin, and melittin		Zarghami et al. (2021)	
Bargavi2022			Pure Ti (grade 2)		Plate: 20 × 10 × 1 mm^3^		Bioglass 45S5	45		24.5		24.5		6		—		Sol–gel		Doped with alumina		Bargavi et al. (2022)	
Bargavi2020			Pure Ti		Plate: 2 × 1 cm^2^		—	—		—		—		—		—		Sol–gel		Composited with zirconia		Bargavi et al. (2020)	
Wu, C. T.2014			Ti-6Al-4V		Plate: 10 × 10 × 2 mm^3^		Sr_2_MgSi_2_O_7_ (SMS) ceramic	—		—		—		—		—		Solid state reaction		—		Wu et al. (2014)	
Catauro, M.2015			Pure Ti (grade 4)		—		Calcium silicate glass coatings containing Ag	—		—		—		—		—		Sol–gel		Doped with different percentages of silver oxide		Catauro et al. (2015)	
Ledda, M.2015			Pure Ti		Plate: 1 × 1 cm^2^		RKKP glass-ceramic composition	43.68		4.53		31.3		11.1		MgO: 2.78, K_2_O: 0.19, CaF_2_: 4.92, La_2_O_3_: 0.5, and Ta_2_O_5_: 1		Aqueous Sol–gel		—		Ledda et al. (2015)	
Mistry2016			Ti-6Al-4V (clinical)		Screw; diameter: 3.5 mm, length: 11 mm and diameter: 4 mm, length: 13 mm		—	59.1		22.24		19.2		5.46		TiO_2_: 1 and B_2_O_3_: 9.4		Melt derived		—		Mistry et al. (2016)	
Soares2018			Titanium (*in vivo*)		Cylinders; diameter: 3.5 mm, length: 7 mm (*in vivo*); disks, diameter: 10 mm (*in vitro*)		BSF18	—		—		—		—		—		—		—		Soares et al. (2018)	
Klyui2021			Ti-6Al-4V		Cylinders; diameter: 2 mm, length: 4 mm (*in vivo*); plate: 20 × 20 × 1 mm^3^ (*in vitro*)		—	32		3.3		39.7		16.3		MgO: 2.7, ZnO: 5, and Al_2_O_3_: 1		—		Composited with HA and doped with Zn, Cu, and Ag		Klyui et al. (2021)	
vanOirschot2016 (a)			Ti-6Al-4V		Plate: 12 × 9 × 1 mm^3^		S53P4	—		—		—		—		—		—		Composited with HA		van Oirschot et al. (2016)	
vanOirschot2016 (b)			Ti-6Al-4V		Cylindrical screw-type; diameter: 4.0 mm, length: 12 mm		S53P4	—		—		—		—		—		—		Composited with HA		van Oirschot et al. (2016)	
Wang2018			Ti-6Al-4V		Disks; diameter: 20 mm, thickness: 1 mm		Bioglass 45S5	45		24.5		24.5		6		—		Melt-quenching		Composited with HA in different ratios		Wang et al. (2018)	
Mehdikhani-Nahrkhalaji, M.2015			Pure Ti (grade 2)		Piece: 20 × 10 mm^2^		58S	57.72		—		35.09		7.1		—		Sol–gel		Composited with poly (lactide-co-glycolide) and hydroxyapatite; composited with poly (lactide-co-glycolide)		Mehdikhani-Nahrkhalaji et al. (2015)	
Popa, A. C.2015			Ti-6Al-4V; pure Ti (grade 1)		Screws; diameter: 3.5 mm, length: 10 mm (*in vivo*); disks, area: 1 cm^2^ (*in vitro*)		—	46.06		4.53		28.66		6.22		MgO: 8.83 and CaF_2_: 5.7		—		—		Popa et al. (2015)	
Zhang2019			Ti-6Al-4V		Cylinders; diameter: 2 mm, length: 6 mm		CaO-MgO-SiO_2_–based bioactive glass-ceramic	49.13		—		43.19		—		MgO: 7.68		Sol–gel		—		Taguchi et al. (2019)	
Newman, S. D.2014			Ti-6Al-4V		Cylinders; diameter: 3.5 mm, length: 6.2 mm		SrBG	—		—		—		—		—		Melt-quenching		Doped with strontium		Newman et al. (2014)	
Wang2020			Ti-6Al-4V		Plate: 5 × 2 × 1 mm^3^		Bioglass 45S5	45		24.5		24.5		6		—		—		Composited with HA and CaSiO_3_		Wang et al. (2020)	
Zhang2021			Ti-6Al-4V (medical grade 23)		Cylinders; diameter: 5 mm, length: 10 mm		—	—		—		—		—		—		Sol–gel		Samples pre-deposited a silica interlayer		Zhang et al. (2021)	
vanOirschot2014			Pure Ti		Cylindrical screw type; diameter: 3.4 mm, length: 10 mm		BG S53P4	—		—		—		—		—		—		Composited with HA		van Oirschot et al. (2014)	
Orita2022			Ti6Al2Nb1Ta0.8Mo		—		AW-GC	—		—		—		—		—		—		—		Orita et al. (2022)	

Ti-6Al-4V, which has been widely applied in orthopedic prostheses and dental implants, is the most widely applied alloy in the included studies. Pure Ti has also been investigated. Bioactive glasses 45S5, S35P5 (Massera et al., 2012), and 58S (Sepulveda et al., 2002) are the most commonly studied basic bioactive glass.

### 3.2 Study type

#### 3.2.1 *In vitro* studies

*In vitro* experiments were introduced in 43 studies, and the cell types tested are listed in Table 3. Among all *in vitro* studies, human osteosarcoma cells, pre-osteoblast MC3T3-E1 cells, mesenchymal matrix cells, stem cells, and fibroblasts have been widely used.

**TABLE 3:** Characteristics of *in vitro* cell experiments.

Cell type		Test aim	Ref.
Human osteosarcoma cell line	U2OS	Impact of biomaterials on osteogenesis and cell reaction mechanism	Shaikh et al. (2019)
	MG-63		Ananth et al., 2013; Ordikhani and Simchi, 2014; Ordikhani et al., 2016; Gaweda et al., 2018; Rastegari and Salahinejad, 2019; Su et al., 2019; Dhinasekaran et al., 2021; Bargavi et al., 2020; Bargavi et al., 2022
	Saos-2		Boschetto et al. (2020)
Pre-osteoblast MC3T3-E1 cell line		Model to investigate osteoblast function	(Fu et al., 2017; Patel et al., 2019; Abushahba et al., 2020; Guimarães et al., 2020; Zarghami et al., 2020; Lung et al., 2021; Nesabi et al., 2021; Safaee et al., 2021; Zarghami et al., 2021)
Fibroblasts cells	NIH3T3 murine fibroblast cells	Possibility of cells to restore tissue injury and regulate bone regeneration	(Catauro et al., 2015; Catauro et al., 2016)
	L-929 mouse fibroblast cell line		(Mistry et al., 2016; Wang et al., 2018; Su et al., 2019)
	Normal human dermal fibroblasts (NHDFs)		Matter et al. (2021)
	Human gingival fibroblast (HGF) cells		Costa et al. (2020)
Mesenchymal stem cell (MSC)	—	Investigate possibility of cell treatment and applications in regenerative medicine	Popa et al. (2017)
	Rabbit adipose derived (rADMSC)		Palangadan et al. (2014)
	Equine adipose tissue derived (ADMSC)		Rau et al. (2020)
	Bone marrow (BMSCs)		Wu et al. (2014)
	Human amniotic mesenchymal stromal cells (hAMSCs)		Ledda et al. (2016)
	Human bone marrow stromal cells (hBMSCs)		Ye et al. (2017)
	Rabbit bone marrow stromal cells (rBMSCs)		Zhang et al. (2016)
Others	Human erythrocyte cells	Biosafety assessment for clinical application	(Bargavi et al., 2020; Dhinasekaran et al., 2021; Bargavi et al., 2022)
	Rat osteoblasts	Stimulate osteointegration	Chen et al. (2014)
	Human umbilical endothelial cell (HUV-EC-C) line	Stimulate blood treatments in early surgery period	Matter et al. (2021)
	Human bone progenitor cell (HBCs)	Stimulate osteointegration	Matter et al. (2021)
	Murine-derived macrophage cell line RAW 264.7 cells	Study mechanism of body immunity protection and regulate process of osteointegration	Wu et al. (2014)
	Osteoclasts (derived from RAW 264.7 cells)	Regulate osteointegration	Wu et al. (2014)
	Caco-2 human colon carcinoma cell line	Investigate cell therapy and strategies for regenerative medicine	Ledda et al. (2015)
	Human dental pulp stem cells (DPSCs)	Possibility of cell therapy	Popa et al. (2015)

Being derived from malignant bone tumors, various types of osteosarcoma cell lines were isolated due to bone tumor categories, for example, U2OS, Saos-2, and MG-63 (Matter et al., 2021). According to their properties of having a higher capacity to help matrix mineralization, being easier to culture, having a more stable phenotype (Rau et al., 2020), and having a faster proliferation rate (Abushahba et al., 2020), they were frequently used for fabricating the osteoblast models. The pre-osteoblast MC3T3-E1 cell line is generated from mouse primary osteoblast culture (Nesabi et al., 2021), which displays similar behavior toward primary osteoblasts and, thus, shows better osteogenic differentiation (Ye et al., 2017). Mesenchymal matrix or stem cells, which are derived from a variety of tissues such as the bone marrow and adipose tissue, are multipotent adult cells (Zhang et al., 2016) and can differentiate into different cell lines. Adipose-derived mesenchymal stem cells (MSCs) are more frequently used because they are abundant and the collection process is simpler and does not cause great trauma (Chen et al., 2014), and this leads to their wide application prospects in regenerative medicine (Palangadan et al., 2014). Such MSCs can also be used to study the osteogenic differentiation potential of glass materials and to study the ability to synthesize certain specific proteins (Rau et al., 2020). Involved in granulation tissue formation, fibroblasts are connective tissue cells that synthesize collagen fibers and matrix components and are essential in the wound healing process (Avcu et al., 2018) and mediation of soft tissue integration (Patel et al., 2019). The test on fibroblasts can illustrate a material's potential application in soft tissue repair.

#### 3.2.2 *In vivo* studies

For animal tests, the characteristics of the *in vivo* studies that were included are listed in Table 4. Many included studies have chosen New Zealand rabbits, and the included surgical sites were the femur, rabbit tibia, and mandible for dogs or pigs. Rabbits are the most commonly used animals due to their size and growth speed. Less soft tissue is found around rabbits' tibia, which is easy to operate on, while the femur has sufficient bone marrow cavity and is, thus, suitable for studying internal fixation of fractures (Guimarães et al., 2020). However, the small size of the bone has led to a reduction in the number and size of implants and, consequently, their style types are also reduced. Pig mandibles have a similar regeneration rate, morphology, and masticatory mechanics to that of humans, and human-sized dental implants are allowed (Zarghami et al., 2020). Even though mini pigs can overcome the problem of being overweight, they are very aggressive and difficult to tame. Dog mandibles are commonly used in dental implant models for assessing bone regeneration around implants (Zarghami et al., 2021). We can use human-sized dental implants in larger dogs that can actively cooperate with rehabilitation treatment programs (Guimarães et al., 2020). However, ethical issues deserve further discussion due to the harm of medical experiments to dogs.

**TABLE 4:** Characteristics of animal experiments.

Study ID	Application field (surgery type)	Basic information about animals						Number (animal numbers/site numbers)		Intervention		Ref.
		Species	Sex	Age	Weight	Surgical site	Substrate material	Control group	Experimental group	Control group	Experimental group	
Zhang2019	Femoral bone defect	New Zealand rabbit	—	—	Approximately 3.0 kg	Femur	Ti6Al4V cylinders; diameter: 2 mm, length: 6 mm	24 (48)	24 (48)	Coated with HA	Coated with CaO-MgO-SiO_2_–based bioactive glass-ceramic	Taguchi et al. (2019)
Newman, S. D.2014	Skeletal reconstructive surgery	New Zealand rabbit	Male	6 months old	3.5–3.8 kg	Distal femur and proximal tibia	Ti6Al4V cylinders; diameter: 3.5 mm, length: 6.2 mm	27 (27)	27 (27)	Coated with HA	Coated with SrBG	Newman et al. (2014)
Zhang2021	Femoral bone defect	New Zealand rabbit	Male	Mature	Average weight of 2.5 kg (SD = 0.3 kg)	Distal femoral condyle	Ti6Al4V porous cylinders; diameter: 5, length: 10 mm^2^, porosity: 68%	6 (12)	6 (12)	Uncoated Ti-6Al-4V	Coated with BG/MBG	Zhang et al. (2021)
Wang2020	Tibial bone defect	New Zealand rabbit	—	6 months old	3–3.5 kg	Tibia	Ti6Al4V plate; 5 mm × 2 mm × 1 mm	—	—	—	Coated with HA/BG/WS composite films	Wang et al. (2020)
Wang2018	Tibial bone defect	New Zealand rabbit	—	—	—	Tibia	Ti6Al4V plate; 5 mm × 2 mm × 1 mm	—	—	HA and BG composite films (90% HA + 10% BG film; 80% HA + 20% BG film; 20% HA + 80% BG film		Wang et al. (2018)
Mehdikhani-Nahrkhalaji, M.2015	Tibial bone defect	New Zealand rabbit	Male	8–10 months	3–3.5 kg	Tibia	Pure titanium screws; diameter: 1.5 mm, length: 6 mm	20 for PBGHA nanocomposite coating, 20 without coating	20	Uncoated pure Ti	Coated with PBGHA nanocomposite/PBG nanocomposite	Mehdikhani-Nahrkhalaji et al. (2015)
van Oirschot,2014	Mandibular implantation	Beagle dogs	—	1–2 years old	10–12 kg	Right side of the mandible	Screw-type pure titanium implants; diameter: 3.4 mm, length: 10 mm	—	16 (48)	—	Coated with different ratios of HA and BG (HA, HABGLow, and HABGHigh)	van Oirschot et al. (2014)
Soares2018	Mandibular implantation	Beagle dogs	Male	Approximately 1.5 years of age	—	Right and left mandible	Morse taper pure titanium implants; diameter: 3.5 mm, length: 7 mm	10 (20)	10 (20)	AE surface implants	Coated with AE surface functionalized with BSF18	Soares et al. (2018)
Klyui2021	Femoral bone defect	Wistar rats	Male	—	240 ± 15 g	Lower femur part	Pure Ti cylinders; diameter: 2 mm, length: 4 mm	Four for each group (24 in total)		1) Uncoated implant; 2) abrasive-surfaced implant with SiC powder; 3) with pure HA; 4) with HA composites; 5) with composite material—BG, 50 wt%; pure HA, 30 wt%; TCP, 20 wt%; 6) with composite material—BG, 65 wt%; HA combination, 35 wt%		Klyui et al. (2021)
Van Oirschot2016	Bone conduction chamber cassette model on the goat transverse process	Dutch Saanen milk goats	—	24 months	60 kg	Spinal transverse	Ti-6Al-4V rectangular samples; 12 × 9 × 1 mm, width: 0.5 mm	10 (40) for each group	10 (40)	Machined Ti, PLD HA, plasma-sprayed HA coating, and biomimetic HA coating	Coated with hydroxyapatite/bioactive glass	van Oirschot et al. (2016)
van Oirschot2016	Iliac bone defect, osteotomies	Saanen goats	Female	24 months	60 kg	Iliac crest	Cylindrical screw-type pure titanium implants; diameter: 4.0 mm, length: 12 mm	8 (32)/8 (32)	8 (32)	Uncoated with grit-blasted/acid-etched surface, coated with hydroxyapatite	Coated with hydroxyapatite/bioactive glass	van Oirschot et al. (2016)
Popa, A. C.2015	Mandibular implantation	Pigs	—	—	—	Mandibular bone	Ti6Al4V dental screws; diameter: 3.5 mm, length: 10 mm	—	—	Uncoated	RF-MS coating with BG/RF-MS BG coating + PDHT	Popa et al. (2015)

#### 3.2.3 Clinical studies

For clinical trials, BG-coated implants are mainly applied in orthopedics and stomatology, and the relative information is shown in Table 5. To date, clinical trials have revealed the effectiveness of BG-coated implants in total hip arthroplasty and dental implantation. In the 10-year retrospective studies carried out by Orita et al. (2022) through clinical evaluation and radiographic assessment after hip arthroplasty surgery, BG-coated implants were proven to have a better survival rate and wear resistance. In the prospective studies by Mistry et al. (2016), by comparing the osteogenesis around dental implants, the BG-coated dental implants contributed to new bone generation.

**TABLE 5:** Characteristics of clinical trials.

Surgery type	Study ID	Study type	Patients				Interventions and comparisons			Outcome indicator	Ref.
			Surgical site	Number	Sex	Inclusion criteria	Substrate material	Control group	Experimental group		
Total hip arthroplasty	Orita2022	Retrospective study	Hip joint	99 patients (116 hips)	—	Received cementless implants, followed up for at least 10 years	Pure titanium hip implants	Uncoated pure titanium	Cementless glass-ceramics containing apatite and wollastonite (AW-GC)	1) Hip joint function; 2) stress shielding; 3) extent of osteolysis; 4) steady-state wear rate; 5) survival rate	Orita et al. (2022)
Dental implant in human jaws	Mistry2016	Prospective clinical trials	Incisor areas of anterior maxilla and mandible	62 patients (126 sites)	35 males and 27 females	Age limited to 18–58 years, and anterior incisor area allows for the same implants; minimal bone requirements of 6 mm in alveolar ridge width and 18 mm in ridge height	Ti6Al4V screw endosseous implant (size: 3.5 and 4 mm diameter × 11 and 13 mm length, respectively)	Uncoated Ti6Al4V; coated with HAp	Coated with BAG (SiO_2_ 59.1%, CaO 19.2%, P_2_O_5_ 5.46%, B_2_O_3_ 9.4%, TiO_2_ 1%, and Na_2_O 22.24%)	1) New osseous tissue deposition; 2) bone loss; 3) plaque index; 4) gingival index; 5) probing pocket depth; 6) gingival recession	Mistry et al. (2016)

## 4 Coating manufacturing technology for BGs on Ti implant surface

### 4.1 Substrate pretreatment

Substrate pretreatment plays an important role in bio-interaction (Pattanaik et al., 2012), which can improve corrosion resistance (Kim et al., 1996) and osteointegration (Buser et al., 2012; Fischer and Stenberg, 2012). Surface roughness can also be increased as well as the adhesion between substrate and coating. Sandblasting is a simple, low-cost method (Zhou et al., 2016). Aluminum oxide and silicon carbon can be injected onto the substrate using high-speed compressed air, which improves the surface roughness. Sandblasting combined with acid etching forms a microporous structure and removes the residual abrasive particles (Pattanaik et al., 2012). Chemical pretreatments are also performed. Alkali and heat-treated implants, which apply hydroxide at high temperatures, form a titanate layer on the Ti surface. This improves the connection between the bone tissue and the implant and enables higher implant stability (Nishiguchi et al., 2003). The porous nanostructures can also increase the bond strength between coatings and substrates (Nesabi et al., 2021). Surface topology is an important surface structure that can effectively regulate the behavior of cells. Numerous studies have shown that rough or micro-/nano-sized topological structures can effectively improve cell behavior, thereby enhancing the integration ability between implants and bone interfaces (Geng et al., 2021a). Micro-arc oxidation can generate a uniform, rough, and porous oxide layer, which contributes to a tighter connection between substrate and coatings (Ma et al., 2016). Among the different coating technologies, polishing and sandblasting the titanium substrate to increase its surface roughness, followed by cleaning the substrate with distilled water, acetone, or ethanol, are the more common pretreatment procedures.

### 4.2 Pulsed laser deposition

**FIGURE 3:**
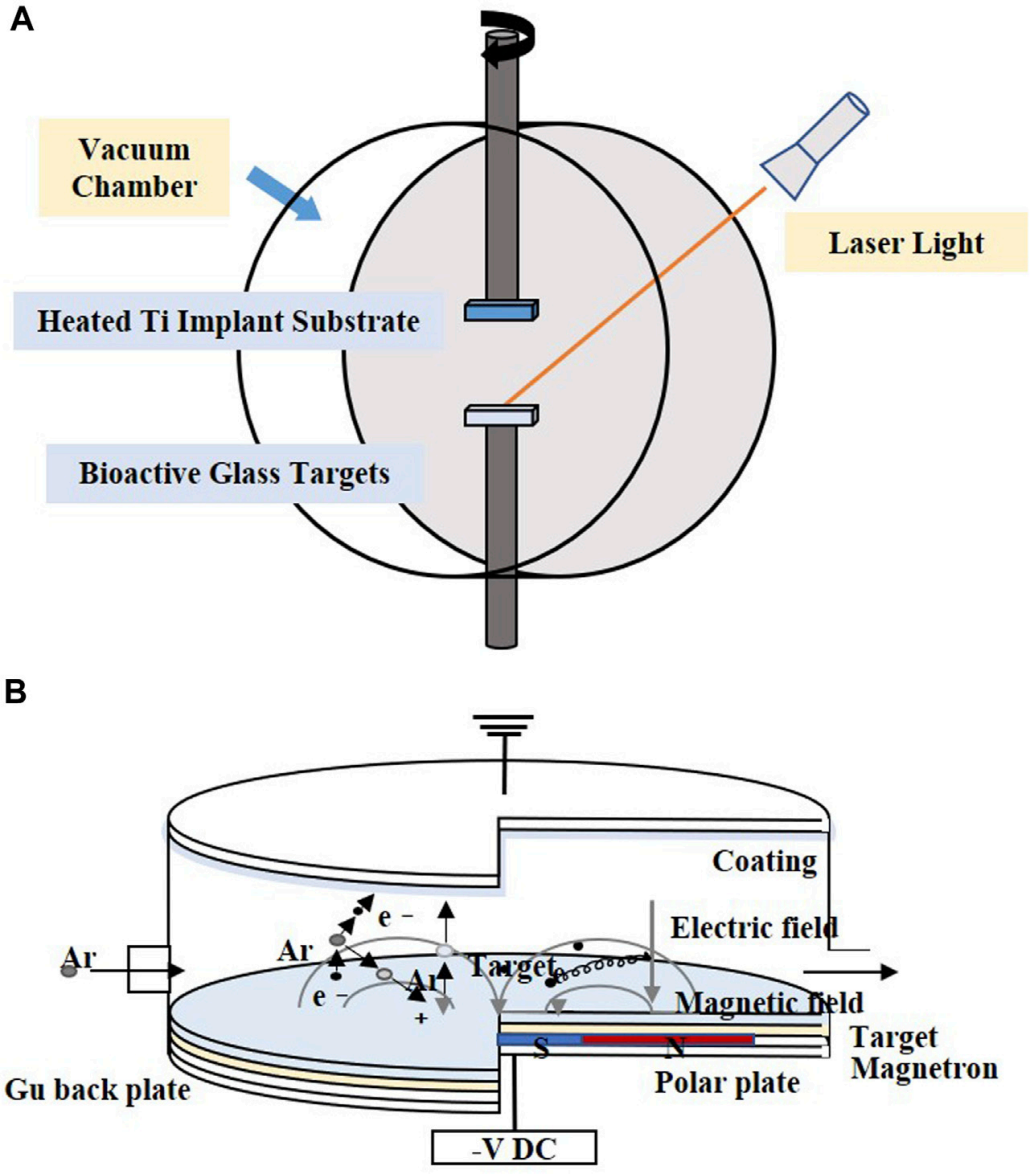
Coating manufacturing technologies of **(A)** PLD and **(B)** magnetron sputtering. The figures were redesigned based on other studies (Torrisi et al., 2007; Calderon Velasco et al., 2016).

PLD is performed under confined conditions. The schematic diagram is shown in Figure 3A. The pulsed molecular laser source can be used to sinter bioactive glass. In a physical coating preparation process, the precise ratio of the coating components can be guaranteed, and the prepared coatings are more uniformly attached. To ensure uniform adhesion and prevent laser single-point substrate surface corrosion, the target is usually coated in a rotary manner (Ma et al., 2016; Wang et al., 2018; Wang et al., 2020).

**FIGURE 4:**
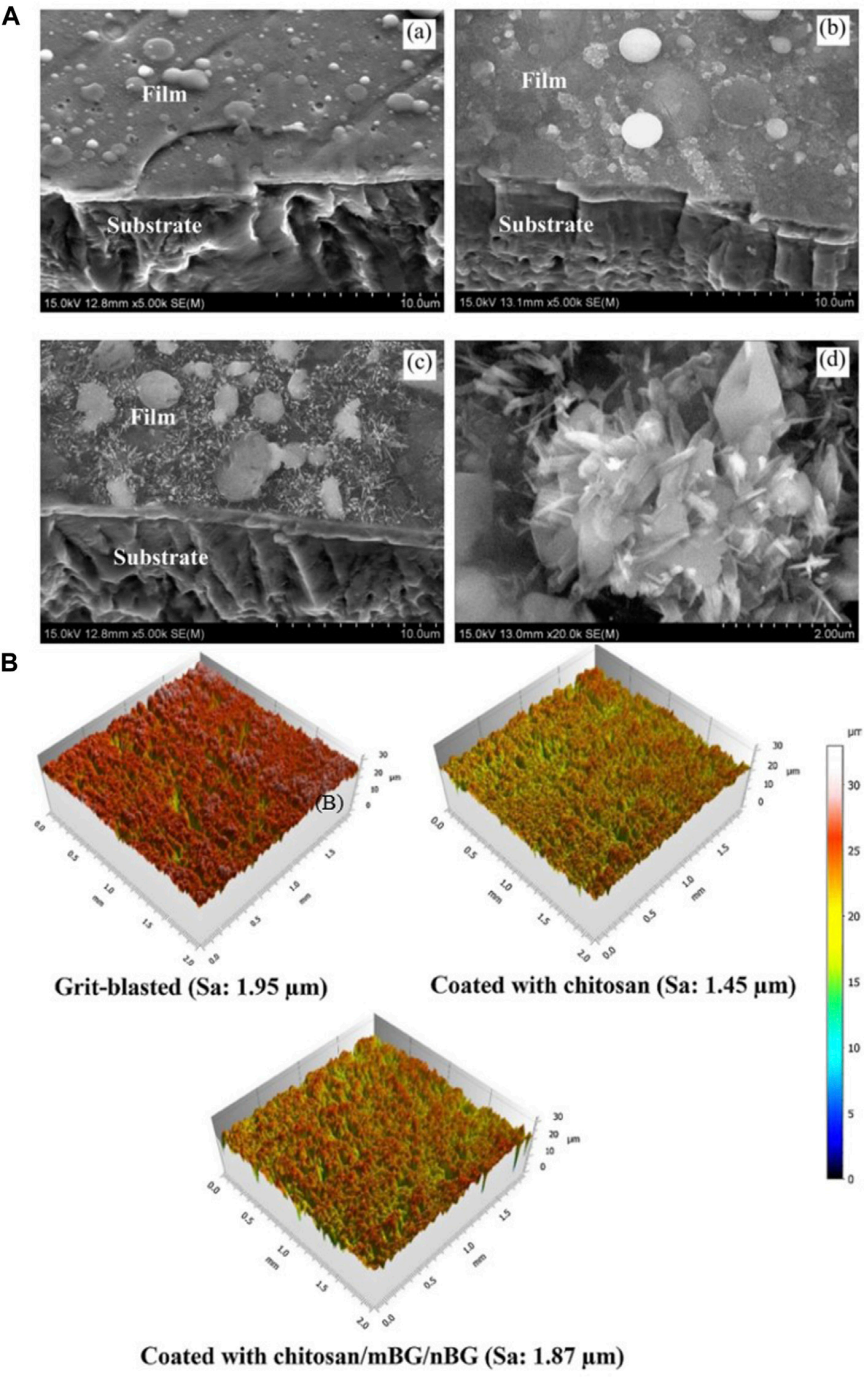
**(A)** SEM morphologies of films under different PLD process parameters: **(a)** 600°C; **(b)** 800°C; **(c,d)** 600°C + 800°C. **(B)** 3D surface topographies of the substrate and coatings. The figures were obtained with permission from Avcu et al. (2018) and Wang et al. (2020).

Table 6 shows the process parameters of the included studies. In the included studies, PLD was usually performed under an environmental temperature of 200°C–800°C. The substrate temperature mainly regulates crystal composition and physiochemical and biological properties by changing the alignments of the target material on the surface and the bonding level of the coating (Figure 4) (Serra et al., 2004; Wang et al., 2020). When the substrate temperature reaches 200°C, the BG coating gains the best mechanical properties and surface appearance and can best bond with the substrates (Serra et al., 2004; Zhao et al., 2008). However, another study has proven that coatings formed under 700°C have the best biological properties (Dhinasekaran et al., 2021). To reduce the overlap between the laser and vapor that it generates, the angle of incidence of the laser projection on the substrate is maintained at 45° (Shaikh et al., 2019).

**TABLE 6:** Parameters for pulsed laser deposition in the included studies.

Study ID	Chamber condition		Laser parameter				Deposition parameter			Ref.
	Gas	Pressure (Pa)	Emission wavelength	Repetition rate (Hz)	Pulse duration (ns)	Base distance (mm)	Deposition time h)	Substrate temperature (°C)	Laser fluence (J/cm^2^)	
Ledda, M. 2016	Vacuum	1.5 × 10^−4^	532	10	10	20	2	500	12	Ledda et al. (2016)
Ma 2016	Vacuum	1 × 10^−4^	1,064	10	7	40	1	200	—	Ma et al. (2016)
Palangadan, R. 2014	Controlled oxygen atmosphere	10^–1^	355	10	—	35	1	400	—	Palangadan et al. (2014)
Wang 2020	Argon gas	45	248	5	—	—	—	600/800	5	Wang et al. (2020)
Wang 2018	Vacuum	3 × 10^−5^	248	5	20	—	1	600	—	Wang et al. (2018)
Ledda, 2015	—	—	532	10	10	20	2	500	12	Ledda et al. (2015)
Shaikh 2019	Vacuum	2 × 10^−3^	532	10	6	50	1	RT to 200	—	Shaikh et al. (2019)
Dhinasekaran 2021	Vacuum	1 × 10^−4^	355	10	—	—	—	200	—	Dhinasekaran et al. (2021)
Rau 2020	Vacuum	10^–4^	532	10	7	20	5	—	12	Rau et al. (2020)

Coatings formed by PLD have a dense surface, a better chemical composition ratio of BG (Ledda et al., 2016), and a microsphere (Palangadan et al., 2014). The nanostructure on the surface can help in implant osteointegration (Rau et al., 2020). PLD mixed with micro-arc oxidation enables the porous morphology of coatings, which results in a better surface appearance and structure (Ma et al., 2016).

### 4.3 Magnetron sputtering

Magnetron sputtering is usually performed under low-pressure conditions, where the deposition chamber is filled with gas, which usually comprises argon (Ar) (Figure 3B). Ar atoms are ionized to produce Ar^+^ and new electrons, with the electronic field accelerating the electrons that bombard the targets and sputter the ions from the target atoms. After deposition, the substrate is heated to optimize the coatings (Shi et al., 2008).

The distance between the substrate and target may influence the deposition rate. A closer distance means a higher deposition rate. However, the increased momentum of the charged particles leads to an increase in the substrate temperature (Maximov et al., 2021). The acceleration voltage, heat treatment time, vacuum pressure, and filling gas also have an impact on the process (Wolke et al., 2005; Popa et al., 2017).

Magnetron sputtering makes the thickness of the coating uniform and adjustable and results in higher adhesion and purity of the coating (Shi et al., 2008), which makes it suitable for covering large areas of the substrate, and the technology is easily scalable to the industrial level under alternating current conditions (Popa et al., 2015).

### 4.4 Dip coating

**FIGURE 5:**
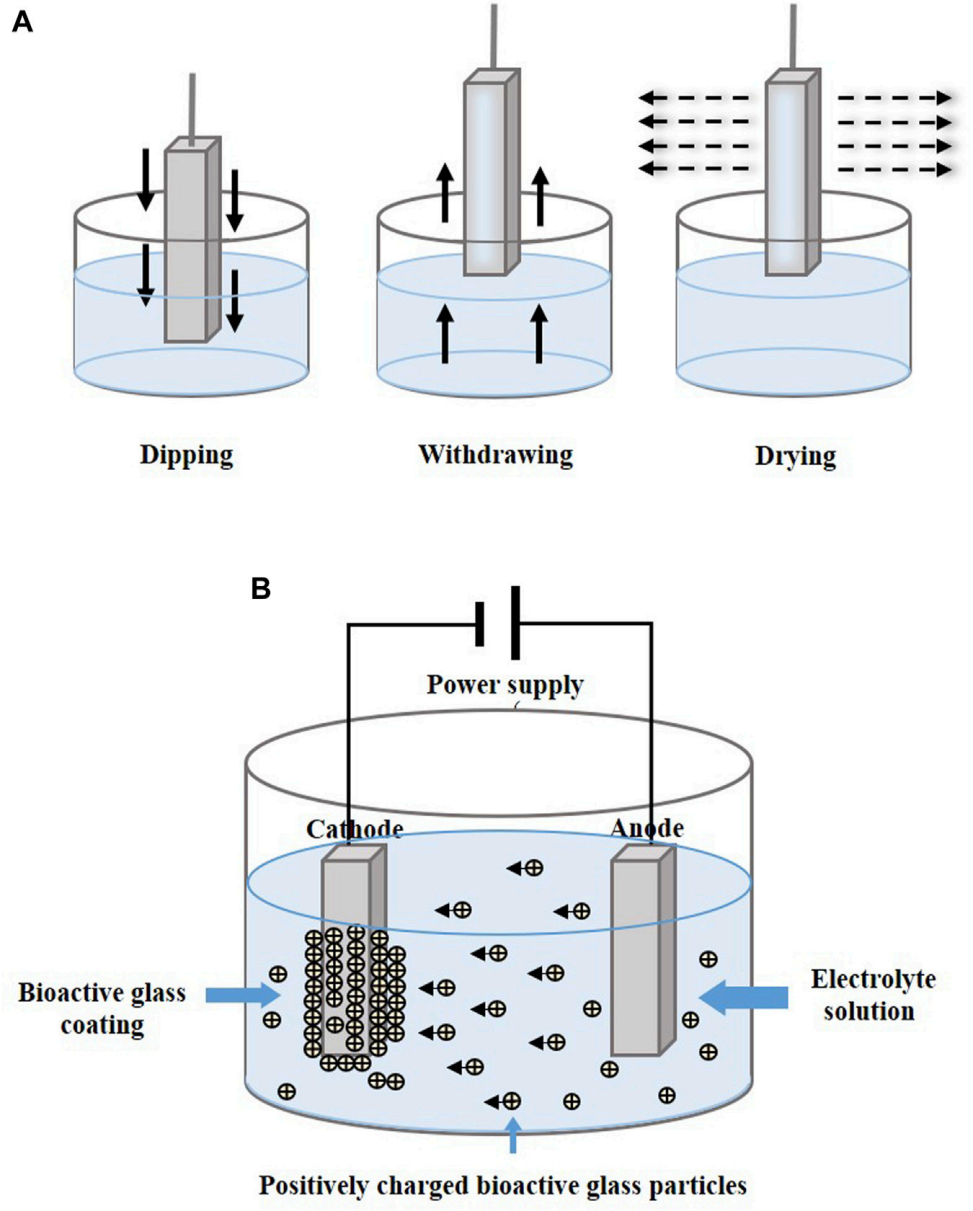
Coating manufacturing technologies of **(A)** dip coating and **(B)** EPD. The figures were redesigned based on other studies (Bohner and Lemaitre, 2009; Boccaccini et al., 2010; Bakhshandeh and Amin Yavari, 2018; Sultana et al., 2021).

Dip coating technology is usually combined with sol–gel, which is a process of preparing bioactive glass (Figure 5A). The glass precursor is obtained from a solution of metal alkoxides and nitrates in ethanol, which is subjected to sufficient hydrolysis and condensation reactions by stirring (Catauro et al., 2016), and the substrates are then immersed in gel. In other cases, the prepared glass is mixed into the solution and infiltrates the substrates. After dipping, drying and calcination are performed to remove excess material and stabilize the coating.

Various conditions such as the extraction speed, dipping times, viscosity of sol, and process of drying influence the coating formation (Brown, 1989). The extraction speed is directly related to the coating thickness. The slower the speed, the thinner the coating and the more the original shape of the substrate can be maintained.

When compared with others, dip coating can be performed under lower temperatures, and the process is cheaper and easier. The coatings are more uniform and have higher purity, which stabilizes the substrate's shape (Fu et al., 2011; Catauro et al., 2016; Fu et al., 2017a). Other components, such as nanoparticles, mesoporous agents, and antimicrobial agents added to the solution, may lead to complications in the coating structure and composition (Tian et al., 2016; Rivadeneira and Gorustovich, 2017).

### 4.5 Electrophoretic deposition

Electrophoretic deposition (EPD) forms a coating by applying a direct current or alternating current electric field between two electrodes, causing charged particles to be dispersed in suspension and move in the direction of the substrate electrode (Figure 5B). The particles are deposited in an orderly manner on the substrate (Wang et al., 2014; Sultana et al., 2021). Heat treatment at 800°C–900°C makes the coatings denser and more stable (Ananth et al., 2013; Khanmohammadi et al., 2020).

Table 7 shows the parameters in the process of EPD in the included studies. The factors affecting the properties of coatings prepared via EPD can be concluded as solutions’ stability, conductivity (Zhitomirsky et al., 2009), and BG powder concentration. The distance between the electrodes, applied electric field voltage, deposition time, temperature, and pH all influence the deposited coatings. Changes in the BG concentration in the solution may increase the electrophoretic mobility, and adjustments made in the deposition parameters can make the coatings denser and more uniform (Jugowiec et al., 2017).

**TABLE 7:** Parameters for electrophoretic deposition in included studies.

Study ID	Coating method	Coating component	Solution	Anode	Cathode	Distance between the electrodes (mm)	Work voltage (V)	Time (min)	Temperature	pH	Coating thickness (µm)	Ref.
Ordikhani, F.2014	EPD	—	Chitosan (0.5 g/L), bioactive glass (0.5 g/L), vancomycin (1 g/L)	Titanium	Titanium	10	15	10	—	3	—	Ordikhani and Simchi (2014)
			Chitosan (0.5 g/L), bioactive glass (0.5 g/L)								55 ± 6	
Ordikhani2016		—	Chitosan (0.5 g/L), bioactive glass (0.5 g/L) in 1 vol% acetic acid in deionized water	Titanium	Titanium	10	15	10	Room temperature	3	—	Ordikhani et al. (2016)
			Chitosan (0.5 g/L), bioactive glass (0.5 g/L), vancomycin in 1 vol% acetic acid in deionized water				15	10				
			Chitosan (0.5 g/L), bioactive glass (0.5 g/L), vancomycin (0.5 g/L, 1 g/L, 1.5 g/L, 2 g/L) in 1 vol% acetic acid in deionized water				10 (for chitosan), 15 (for chitosan/bioactive glass composite)	20			128	
Ananth2013		(Ca Mg)_3_(PO_4_)_2_, CaSiO_3_, ZrO_2_	1) 5 wt% YSZ [yttria-stabilized zirconia (YSZ)], 2) 1YSZ-2BG (5 wt% YSZ-10 wt% BG) in isopropanol [bioactive glass (BG)]	Platinum	Ti6Al4V	10	70	5	Room temperature	—	1) 4–5, 2) 12–15	Ananth et al. (2013)
			1) 10 wt% YSZ, 2) 2YSZ-2BG (10 wt% YSZ, 10 wt% BG) in isopropanol									
Patel2019		Mesoporous bioglass nanoparticles, chitosan	Mesoporous bioglass nanoparticles (0.25 g/L), chitosan (0.50 g/L)	Stainless steel	Pure titanium	10	25	1–5 min	—	3.5	—	Patel et al. (2019)
			Mesoporous bioglass nanoparticles (0.50 g/L), chitosan (0.50 g/L)									
			Mesoporous bioglass nanoparticles (0.75 g/L), chitosan (0.50 g/L)									
Avcu2018		—	Chitosan (0.5 g/L), micro-45S5 bioactive glass (0.5 g/L), nano-bioglass (0.5 g/L), acetic acid (1 vol%), deionized water (20 vol%), ethanol (79 vol%)	Ti6Al4V alloy	Ti6Al4V	10	4, 6, 8, 10, 15, 20	3	Room temperature	4–5	—	Avcu et al. (2018)
			Chitosan (0.5 g/L), nano-bioglass (0.5 g/L), acetic acid (1 vol%), deionized water (20 vol%), ethanol (79 vol%)									
			Chitosan (0.5 g/L), micro-45S5 bioactive glass (0.5 g/L), acetic acid (1 vol%), deionized water (20 vol%), ethanol (79 vol%)									
Costa2020	PEO	SiO_2_, CaO, CaCO_3_, Na_2_O, P_2_O_5_	C_3_H_7_Na_2_O_6_P (0.0010 M), Na_2_SiO_3_-5H_2_O (0.014 M), C_4_H_6_O_4_Ca (0.20 M), NaNO_3_ (0.50 M), C_3_H_7_Na_2_O_6_P (0.0010 M), Na_2_EDTA·2H_2_O (0.025 M)	Pure Ti	Steel	—	500	7	23.0°C ± 1.5°C	—	23.42282	Costa et al. (2020)

### 4.6 Hydrothermal deposition

Hydrothermal deposition is a multiphase reaction of dissolution and recrystallization of materials under high temperature and pressure with an aqueous solution as the reaction system in a closed autoclave, leading to the formation of precipitates (Pore et al., 2021). This method can be used for the preparation of powders and as a coating technique for materials.

The process essentially involves attaching thermocouples and pressure sensors to the reactor assembly, setting the parameters, and heating the reactor. Subsequently, pressure builds up and the coating is deposited on the sample in a supercritical environment (Ali et al., 2018).

The method is simple and inexpensive and can be used to synthesize coatings with uniform thickness, orientation, and shape directly in an aqueous solution (Valanezhad et al., 2015). The coating structure can be controlled by changing the synthesis parameters. However, with this method, it is not easy to control the crystal structure formed, and due to the environmental requirements of high temperature and high pressure, it is more dependent on the experimental equipment.

## 5 Biocompatibility and antibacterial properties

### 5.1 Cytotoxicity and cell activity

Cytotoxicity is the killing of cells by chemicals without involving the cellular mechanisms of apoptosis or necrosis. After implantology, ions that reach the cytotoxic concentration leach out of the glass coating and interact with the cells (Al-Noaman et al., 2012). As an important indicator to assess the safety of biological materials, cytotoxicity is assessed by *in vitro* studies that simulate the survival environment of cells. A lower cell survival rate indicates higher cytotoxicity, indicating clinical risk (Wataha et al., 1994). Cell viability above 70% is usually considered non-cytotoxic (Wei and Ding, 2017). The release of large amounts of alkaline ions can have adverse effects on living cells. The increase in pH at the implantation site is accompanied by the dissolution of bioactive glass, which increases by-products and ultimately leads to toxic effects on the surrounding tissues (Jones, 2013). The adverse effect causes tissue reactions such as inflammation, necrosis, induction of immunity, and carcinogenesis (Costa, 1991).

Cytotoxicity is influenced by the substance and doped content in the glass coating. BG doped with silver ions, cobalt oxide, and titanium dioxide has shown that the doping of cobalt oxide causes higher cytotoxicity than that of silver and titanium dioxide (Lung et al., 2021). This is mainly because cobalt ions induce oxidative stress and activate intracellular nicotinamide adenine dinucleotide phosphate (NADPH) oxidase to produce ROS, which causes oxidative damage to cells (Chattopadhyay et al., 2015) and affects cell morphology and viability (Fleury et al., 2006). A high level of silver in the coating increases the level of released nitrate and improves its cytotoxicity (Catauro et al., 2015).

Cell activity refers to the ability of cells to maintain or resume normal physiological activities, such as cell adhesion, proliferation, migration, differentiation, and metabolism (Patel et al., 2019), which is affected by environmental factors, such as cell culture parameters, attached drugs, and growth factors. Cell adhesion refers to the cellular ability to contact and bind to adjacent cells or the extracellular matrix (ECM) (Humphries et al., 2009). Cell spreading is the behavior of cells on the surface of a biomaterial, which is influenced by certain protein molecules (Cuvelier et al., 2007). Cell proliferation is the process of cell division by DNA replication and other reactions under the action of cycle regulators, resulting in an increase in cell numbers, which is the basis for normal tissue development and maintenance (Xynos et al., 2001). Cell migration is the movement of cells after receiving a certain signal or concentration gradient of a substance and is essential for proper immune response and wound repair (Trepat et al., 2012). The morphological and functional changes that occur because of the selective expression of cellular genes are defined as cell differentiation (Ponzetti and Rucci, 2021).

**FIGURE 6:**
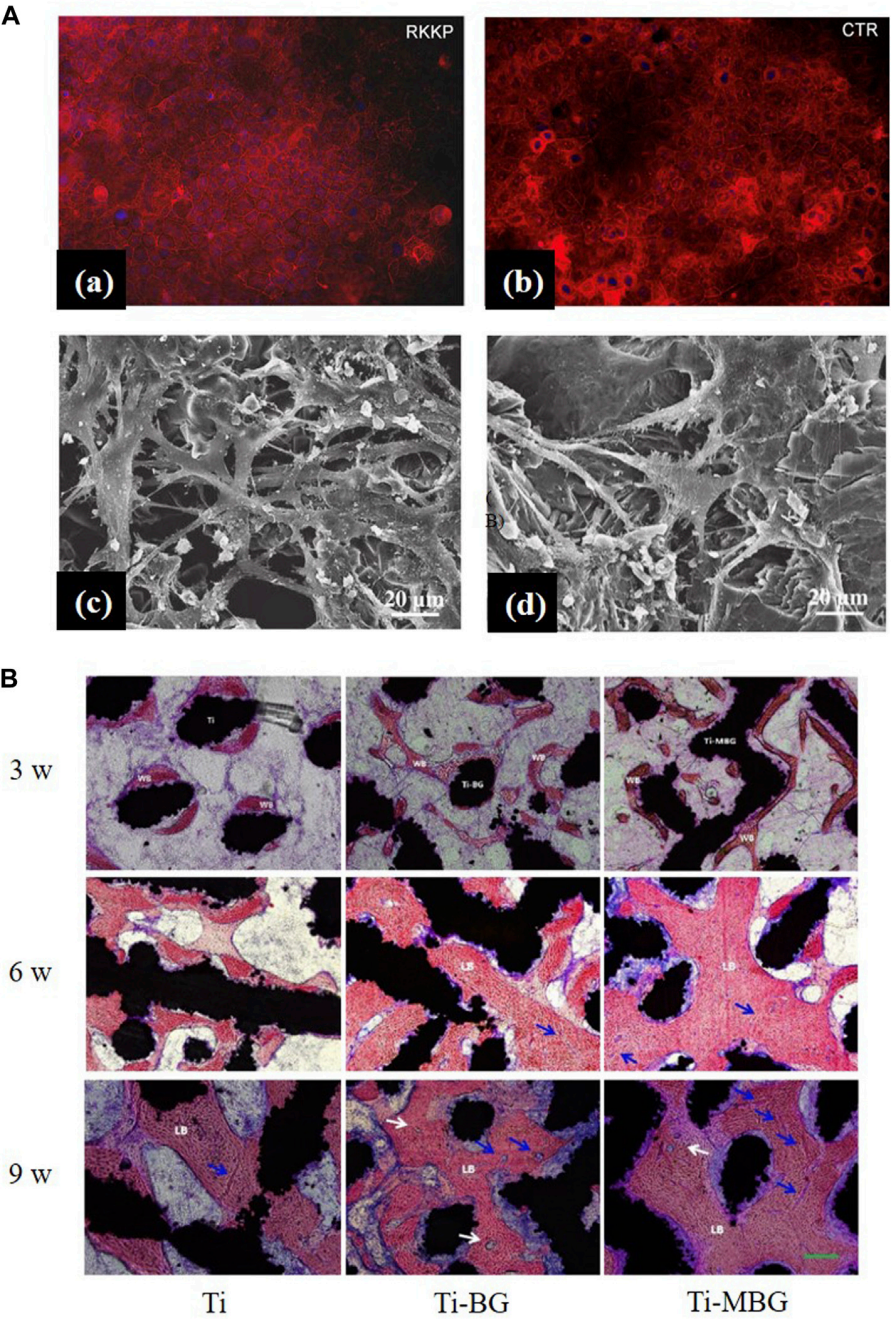
**(A)**, **(a,b)** Actin distribution in the Caco-2 cell line: cells seeded on the RKKP film surfaces and plastic Petri dishes (CTR: critical temperature resister, a semiconductor ceramic material). **(c, d)** Morphological aspects of MG-63 osteoblast cells cultured on YSZ-BG-coated Ti6Al4V. **(c)** Cells on 1YSZ-2BG are similar, but the osteoblasts are well spread and present cytoplasmic extensions forming a continuous surface layer. **(d)** Cell morphology on 2YSZ-2BG coating is similar to **(c)**, but filopodial extensions are fewer. The figures were obtained with permission from Ananth et al. (2013) and Ledda et al. (2015). **(B)** Representative histological images of non-decalcified sections are obtained by methylene blue acid fuchsin staining. Blue arrows, neovascularization; white arrows, the Haversian system; WB, woven bone; LB, lamellar bone. The figures were obtained with permission from Zhang et al. (2021).

BG 45S5 is generally regarded as the gold standard for bioactive glass, and its ionic lysate induces adhesion and proliferation of cells (Abushahba et al., 2020). Calcium silicate-based materials release calcium and silicate ions, which induce osteoblast proliferation by gene activation (Catauro et al., 2016). Uniform coatings give the best cell metabolic, whereas inhomogeneous coatings, where some cells are in direct contact with the Ti matrix, are less biocompatible (Catauro et al., 2016). However, studies have shown that, when compared with HA coatings, BG-coated samples lead to the rupture and contraction of cells (Dhinasekaran et al., 2021). The surface roughness and profile of the coatings influence cell adhesion and proliferation (Gaweda et al., 2018). Fluorescence staining was performed on cells to observe the effect of BG coatings on the distribution of cytoskeleton, a typical sub-apical localization of the cytoskeleton around the cell membrane can be found. This phenomenon confirms the adhesion and proliferation ability of the cells and demonstrates that BG coatings contribute to the differentiation of the Caco-2 cell line [Figure 6A(a,b)] (Ledda et al., 2015). Doping zinc oxide into BG 45S5 stimulates osteoblasts proliferation and, thus, improves the combination between the implants and bone tissue (Ishikawa et al., 2002; Oki et al., 2004). BG-coated implants doped with Ag_2_O increase cell viability. A large number of cells can be observed on the surface with Ag_2_O in 0.008 % mol/mol, which may have resulted from the release of nitrate ions (Catauro et al., 2015). On the surface of composite coatings comprising chitosan and BG, more cell adhesion, a higher rate of proliferation, and an extended and expanded cytoskeleton can be observed (Patel et al., 2019; Zarghami et al., 2021). On the surface of yttria-stabilized zirconia (YSZ)-BG composite coatings, a large coverage of osteoblasts can be observed, with many filamentous adhesions between the cells and visible nodule formations, which is an early feature of cell differentiation [Figure 6A(c,d)]. However, increasing the relative content of YSZ in the coating decreases the cell activity, as more yttria Y^3+^ ions are released (Ananth et al., 2013).

In *in vivo* experiments, after the formation of HA on the surface, five stages of biological events occurred: 1) growth factors adsorption, 2) bone progenitor cells adhesion, 3) proliferation, 4) differentiation, and 5) production of the extracellular matrix, which enhances bone healing (Popa et al., 2015). The surface roughness contributes to a larger cell attachment area. It has been observed that cell density and bone healing in implants with rough surfaces are significantly better than in those with smooth surfaces (Klyui et al., 2021). Dissolved ions such as Ca, Mg, and Si can activate the expression of bone-related genes by regulating bone-related cell growth and metabolism (Taguchi et al., 2019; Zhang et al., 2021). The wettability and hydrophilicity of the surface also promote cell proliferation (Soares et al., 2018).

### 5.2 Hemocompatibility analysis

Hemocompatibility is the ability of blood to tolerate a material without causing significant adverse blood reactions when the material is in contact with blood (Nalezinkova, 2020). The main adverse blood reactions involve thrombosis. The absorbance of blood proteins on the surface of the materials triggers a series of cascade reactions, resulting in thrombosis, and the coagulation cascade spreads rapidly, leading to death in severe cases (Manivasagam et al., 2021). Hemolysis, which reflects hemocompatibility, is caused by adverse reactions to any toxic substance that comes in contact with blood (Dhinasekaran et al., 2021). The percentage of hemolysis (hemolysis%) is calculated on the basis of the following formula:

$$\text{Hemolysis}\%=\frac{\text{free}\;\text{hemoglobin}\;\text{concentration}}{\text{total}\;\text{hemoglobin}\;\text{concentration}}\times 100\%.$$

If the hemolysis% of a sample is <2%, it is non-hemolytic; a hemolysis% between 2% and 5% indicates that it is slightly hemolytic; if the hemolysis% >5%, it is considered hemolytic. Materials that are blood compatible are considered to have a hemolysis% less than 5%.

Studies have demonstrated that the hemolysis% of BG powders in all concentrations is lower than that of HA at the same concentrations, while more red blood cells (RBCs) are ruptured with BG coating. This may be due to the release of sodium ions from the BG coating, causing RBCs to rupture and, thus, exhibit hematotoxicity, which is corrected by washing after coating (Durgalakshmi et al., 2020; Dhinasekaran et al., 2021). Bargavi et al. (2022) found that BG coatings doped with various concentrations of alumina (Al) exhibited non-hemolytic properties and improved hemocompatibility when compared to pure BG coatings, where BG coatings doped with 10% Al had the best hemocompatibility. The hemocompatibility of BG coatings doped with zirconia (Zr) has also been investigated. With an increase in Zr concentration, the hemolysis% of the coating slightly decreased; while BG coatings doped with 5% and 10% Zr showed non-hemolysis, BG coatings doped with 15% Zr showed slight hemolysis (hemolysis % <2.5%) (Bargavi et al., 2020). Generally, BG-coated implants show great hemocompatibility.

### 5.3 Anti-inflammatory properties

Inflammation is an immune response of the body to resist harmful irritation, which helps maintain tissue homeostasis during injury or infection (Medzhitov, 2010; Wu et al., 2019; Chang and Xiong, 2020); however, excessive inflammatory responses form fibrous capsules that prevent implants' osteointegration. Therefore, superior anti-inflammatory property is critical for implant success.

**FIGURE 7:**
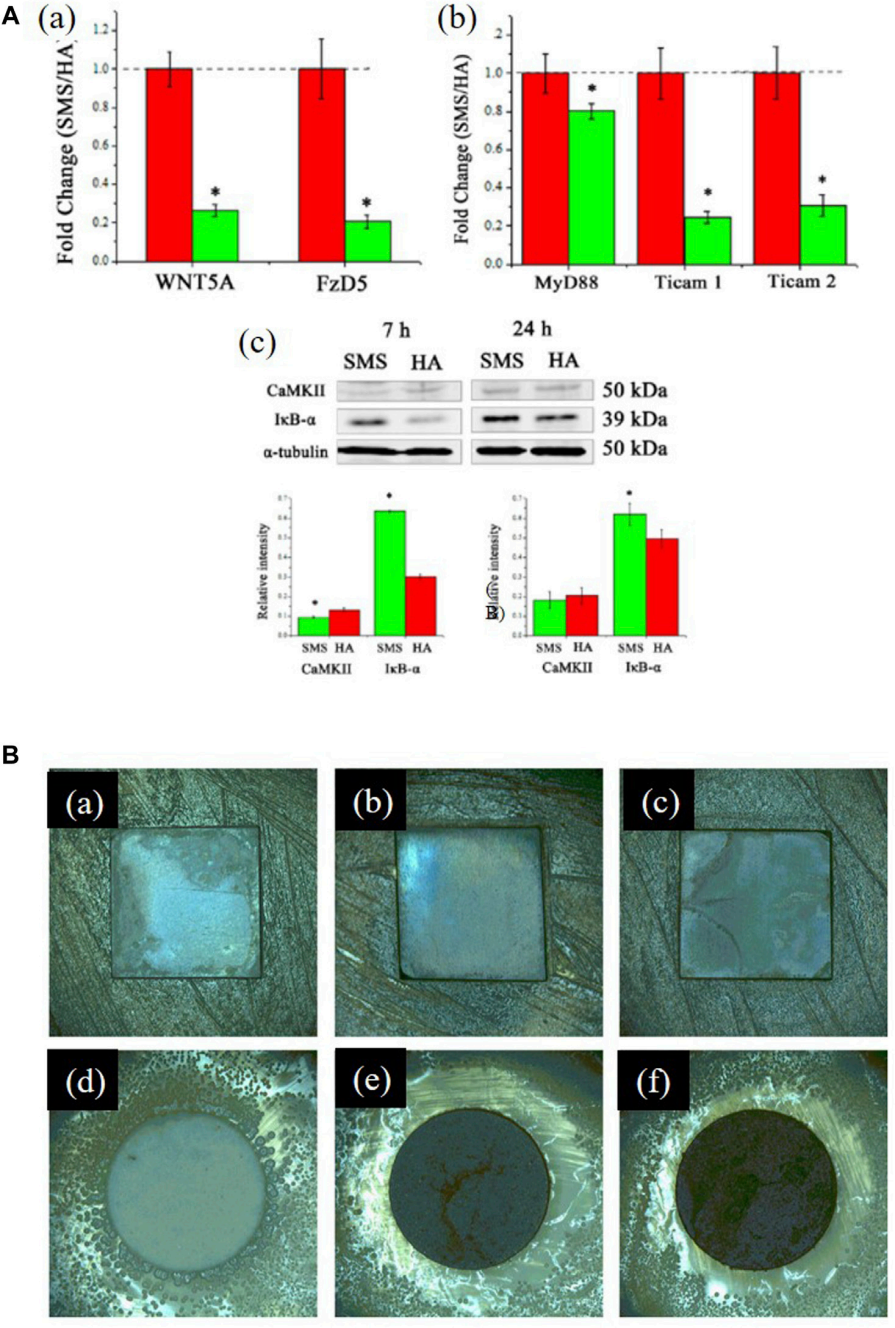
**(A), (a)** Fold changes of WNT5A/Ca2+ pathway-related genes: WNT5A and Fz5. **(b)** Fold changes of Toll-like pathway-related genes: MyD88, TICAM1, and TICAM2. **(c)** Western blotting analysis of CaMKII and IκB-α expression. *significant difference by comparing RAW 264.7 cells cultured in SMS coating with HA (*p* < 0.05). The figures were obtained with permission from Wu et al. (2014). **(B)** Typical optical images of antibacterial test results of the coated samples against *Staphylococcus aureus*: **(a)** 0 Ag, **(b)** 5 Ag, **(c)** 10 Ag, and **(d–f)** their corresponding glass wafer samples: 0 Ag, 5 Ag, and 10 Ag: Ag/Ca atomic ratios of 0%, 5%, and 10%, respectively. The figures were obtained with permission from Fu et al. (2017).

There is no significant difference in the expression of anti-inflammatory factors in human amniotic mesenchymal stromal cells (hAMSCs) on RKKP glass-ceramic coating when compared with the control group, which indicates that the coating did not affect the expression of hAMSCs' anti-inflammatory factors (Ledda et al., 2016). Wu et al. (2014) found that bioactive Sr_2_MgSi_2_O_7_ (SMS) ceramic coatings exhibited superior anti-inflammatory effects compared to HAp coatings, and their mechanism of inhibiting the inflammatory response may be due to the 1) inhibition of the Wnt5A/Ca^2+^ pathway, which enhances the inflammatory response by decreasing the Ca^2+^ concentration or 2) inhibition of inflammatory cytokine expressions by the Toll-like receptor (TLR) pathway, which induces an immune response by the release of Mg^2+^ and Sr^2+^ (Figures 7A a–c).

For *in vivo* experiments, the inflammatory response of the host to the implant is a normal bodily reaction, often manifested as a local inflammatory response and vascular congestion, which disappears after some time (Taguchi et al., 2019). The gingival index determines the inflammatory status by observing the gingival condition, while the periodontal pocket is a manifestation of the pathological inflammatory response (Löe, 1967; Donos, 2018). In clinical trials, in follow-up survey statistics, the gingival index and depth of periodontal pockets were smaller in BG-coated groups, which showed a higher success rate of implantology (Mistry et al., 2016).

### 5.4 Bioactivity properties

#### 5.4.1 Osteointegration

Osteointegration, also known as osseointegration, mainly describes the level of direct connection between an artificial implant and bone tissue without an intermediate fibrous connective tissue layer (Brånemark et al., 1977; Goriainov et al., 2014). A good interface between the implant and bone is an important factor in the formation of dense new bone and, thus, for the osseointegration of both (Agarwal et al., 2015; Hu et al., 2019; Sang et al., 2022). The wettability and surface energy of a material can change the binding of implants to osteoblasts after implantation (Chen et al., 2011).

In animal experiments, BG coating improved implant wettability and enhanced cell viability in the early stages of bone healing, thus significantly increasing bone-to-implant contact (BIC) and bone mineral density (BMD) (Soares et al., 2018), with new bones being formed around the implant and closely combined with the bone tissue (Taguchi et al., 2019). Strontium-substituted bioactive glass (SrBG) coating can stimulate bone formation by releasing dissolved products, showing a superior bone fixation effect (Newman et al., 2014). Reparative osteogenesis formed around the BG/HA/TCP composite coating implants (Klyui et al., 2021), and a perfect fusion with the bone tissue could be observed around the HA/BG/wollastonite (WS) composite coating implant (Wang et al., 2020). However, some studies have also found that in HA/BG coatings, when the concentration of BG is increased, a faster dissolution rate of BG leads to new bone damage and limits the combination of implants and bone tissue (Wang et al., 2018). Only BG containing a certain weight percentage, that is, 40–60wt% SiO_2_, can promote osteogenesis (van Oirschot et al., 2014).

In clinical trials, good bone ingrowth can be found near the implants in BG-coated hip implants (Orita et al., 2022). The bone regeneration around the oral implants is better, with osteoid formation and increased mineralization, which is specifically reflected in the higher median interface density (MID), discrete interface density (DID), and interface radiodensity (IFD) observed at 6 months (Mistry et al., 2016).

#### 5.4.2 Osteogenesis

Osteogenesis refers to bone tissue formation, which is a complex procedure of osteo-development. Bone matrix mineralization and secretion are eternal procedures controlled by osteoblasts (Popa et al., 2015). Based on the included studies, osteogenesis mainly reflects on apatite formation in simulated body fluid (SBF), positive osteoblasts response, and rapid increase in new bone formation and mineralization *in vivo*.

##### 5.4.2.1 Apatite formation in SBF

BG and doped ions in the coating significantly affect apatite formation in SBF. Phosphate in BG can promote apatite formation in SBF (Li et al., 2021). Mesoporous bioactive glass (MBG) coatings with an ordered mesoporous structure exhibit more evident apatite deposition than BG coatings (Zhang et al., 2016).

**FIGURE 8:**
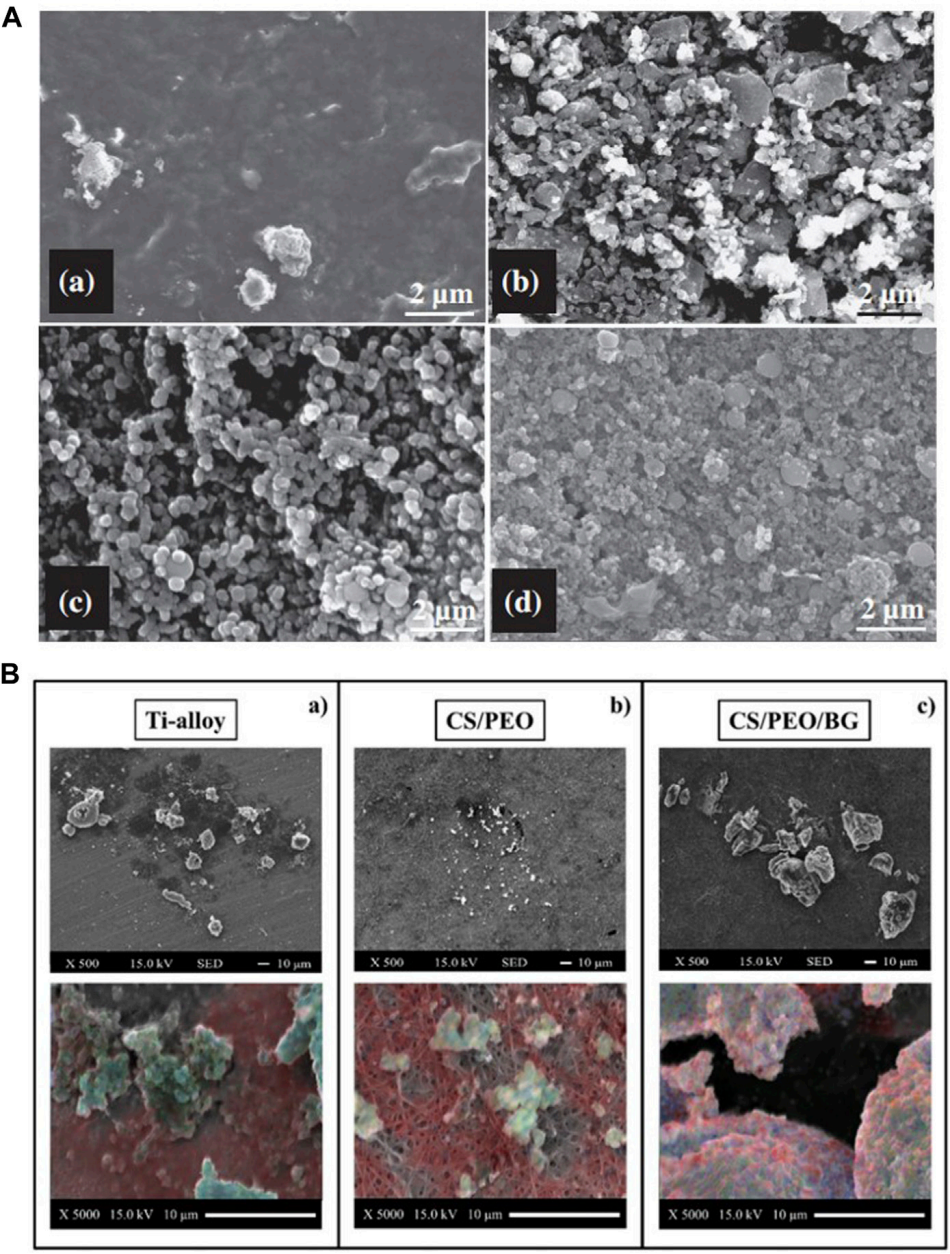
**(A)** SEM images of the 1YSZ-2BG–coated Ti6Al4V alloy, which was immersed in SBF for various time durations (days): **(a)** 3, **(b)** 7, **(c)** 14, and **(d)** 21. **(B)** SEM images of bone formation on the three substrates and EDX elemental maps labeled calcium (blue), carbon (red), and phosphorous (blue) to identify the presence of the mineral. The figures were obtained with permission from Ananth et al. (2013) and Boschetto et al. (2020).

Ananth et al. (2013) observed more calcium phosphate particle deposition by increasing the relative content of BG in YSZ-BG composite coatings, which may be due to the promotion of apatite nucleation by the Si–OH group in the BG coating (Figure 8A). Increasing the BG content in chitosan/BG composite coatings also enhances the osteo-biological activity of the coating (Avcu et al., 2018). A high SiO_2_ content reduces the dissolution rate of BG and influences surface apatite formation, which indicates that the formation of surface apatite can be improved by reducing the content of SiO_2_ and doping an appropriate amount of Ag and Co (Lung et al., 2021). However, Ag^+^ is smaller in size than Ca^2+^ and binds more firmly with unbridged oxygen, so high Ag content is not conducive to the formation of HA (Catauro et al., 2015). The BG-Al composite coating was created using the sol–gel method, and with an increase in Al concentration, the growth rate of apatite accelerated (Bargavi et al., 2022).

##### 5.4.2.2 Osteocyte experiments

A material's influence on cellular behavior is essential in osteogenesis. The connection between the bone *in vivo* has two steps: first, the generation of a carbonate HA layer on the glass surface, to which the osteogenesis-related cells will subsequently respond. The dissolution products of BG modulate the proliferation and differentiation of cells to accelerate the integration of bone (Crush et al., 2021). *In vitro* cellular experiments reveal the production of intracellular osteogenic markers and assess the osteogenic activity of BG. The maturation of osteoblasts is categorized into proliferation, differentiation, and mineralization (Owen et al., 1990). In the proliferative stage, cells express genes that regulate the cell cycle and growth and form the ECM. Alkaline phosphatase (ALP), which is expressed in the initial differentiation stage, and osteopontin (OPN), which is expressed in the initial mineralization stage, induce matrix maturation and mineralization, and their increased expressions in osteoblast markers promote mineral deposition (Saino et al., 2010).

Ions released by BG dissolution can stimulate gene expression (Hench et al., 2020), which indicates that glass components have a significant impact on cells’ proliferation and differentiation. Si and Ca groups play a more important role in osteo-associated cells’ differentiation and proliferation than P and Na groups (Su et al., 2019). Cell inoculation on BG-coated surface significantly increased the content of mineralized matrix deposition (Figure 8B) (Boschetto et al., 2020), markers of early differentiation such as ALP and RUNX2, and expression of osteocalcin (OCL), which is expressed in late differentiation (Ledda et al., 2016). It has also been shown that the mesoporous physical structure of glass promotes the effective release of Ca and Si ions, leading to a higher level of cell differentiation (Zhang et al., 2016; Ye et al., 2017). When compared with uncoated Ti and BG-coated implants, more collagens were released in osteoblasts co-cultured with zirconia-containing BG-coated implants because the BG-Zr composite mediates collagen synthesis by changing the pH through an ion-release mechanism (Bargavi et al., 2020). Meanwhile, a higher pH also favors bone formation processes, which include the cross-linking of collagen chains and subsequent deposition of HA (Wang et al., 2011; Bargavi et al., 2020).

##### 5.4.2.3 *In vivo* trials

Osteogenesis in clinical trials contributes to the complete restoration and biomechanical properties of natural bone (Santos et al., 2009). Mechanical stabilization and appropriate host response are essential to ensure osteogenesis (Lavenus et al., 2015).

The intrinsic mechanism of osteogenesis is inextricably linked to the ion release process of the BG coating. The silica-rich layer on BG coatings leaches into the local tissue fluid, regulating the osteoblast cycle and allowing rapid osteogenesis and mineralization, which increases peri-implant osteogenesis and mineralization (Xynos et al., 2000; Hench and Greenspan, 2013). Ion dissolution products containing Ca, Mg, and Si bioactive glasses activate the expression of bone-related genes such as bone morphogenetic proteins (BMPs) and vascular endothelial growth factor (VEGF), stimulating osteogenesis and angiogenesis (Huang et al., 2009; Sun et al., 2009; Gu et al., 2011; Hoppe et al., 2011; Saffarian Tousi et al., 2013; Zhai et al., 2013; Henstock et al., 2015; Shamsi et al., 2017; Naseri et al., 2018; O’Neill et al., 2018; Zafar et al., 2019; Zhou et al., 2019).

In animal tests, BG-coated implants show active areas on the surface that serve as the origin of osteogenesis, forming bones with better morphology, maturity, quantity, and thickness than in control groups, and with more rapid and effective osteogenesis (Figure 6B) (Taguchi et al., 2019; Zhang et al., 2021). It has also been shown that a more mature new bone was observed around BG-coated implants in the early stages of bone healing, but as the healing time increased, the advantage of BG-coated implants was no longer evident, since surface roughness affects the adhesion, migration, and differentiation of osteoblasts and thereby the osteogenesis process (Soares et al., 2018).

Poly (co-glycolide propionate)/BG (PBG) nanocomposite coatings induce more than 85% in bone formations. On HA/BG/WS composite coatings, the formation of surface bone tissue has been observed (Wang et al., 2020), while BG composite coatings with 30 wt% HA (pure) and 20 wt% TCP (tricalcium phosphate) show a larger area of bone formation around the implant (Klyui et al., 2021). Furthermore, the peri-implant osteogenic capacity may be related to the implant site and animal species, and the bone quality and quantity at the implant site may interfere with possible significant differences between experimental groups (van Oirschot et al., 2014).

##### 5.4.2.4 Clinical trials

In clinical trials, BG-coated groups show a higher success potential: the least marginal bone loss (MBL), the highest IFD in both low- and high-density bone tissue, and a better deposition and mineralization of new bone tissue around the implant (Mistry et al., 2016). Furthermore, BG-coated hip implants have better bone growth conditions with excellent survival rates and wear resistance (Orita et al., 2022).

#### 5.4.3 Angiogenesis

Angiogenesis, also known as vascular regeneration, is the sprouting and remodeling of neovascularization in the original vascular network (Huang and Nan, 2019), which is essential for tissue repair after implantation (Mehdikhani-Nahrkhalaji et al., 2015; Nowak-Sliwinska et al., 2018; Klyui et al., 2021). The formation of vessels helps transport oxygen, nutrients, minerals, and osteoprogenitor cells over long distances, contributing to bone healing (Zhang et al., 2021). Cerium oxide and BG doped with 2% Zn nanocomposite coatings exhibit superior pro-vascular regenerative capacity and the mechanism is related to the regulation of the hypoxic response and structural reorganization of cells (Matter et al., 2021). Increasing the porosity of BG-coated porous scaffold, reducing the thickness of the coating, and improving the surface roughness all provide space for new bone formation, contributing to angiogenesis (Zhang et al., 2021).

#### 5.4.4 Soft tissue adhesion

After implantation, soft tissue reaction leads to the formation of a fibrous capsule, which contacts the implant without adhesion, allowing relative movement between the implant and the surrounding tissue. Appropriate soft tissue adhesion holds the implant stably in the surrounding tissue (Lee et al., 2010), which limits hematoma and abscess formation and prevents infection (Lee et al., 2010; Zigterman et al., 2019).

Surface roughness, coating composition, and structural design of the implants are the main factors affecting soft tissue adhesion (Lee et al., 2010; Zigterman et al., 2019). The ceria and BG doped with 2% Zn nanocomposite coatings reduce the biomineralization behavior to adapt to soft tissue while inducing the generation of vascular endothelial cells without cytotoxicity to gingival fibroblasts. It can promote rapid wound healing and exhibit superior soft tissue regeneration abilities in subsequent scratch assays (Matter et al., 2021). During oral implantology, the surrounding blood clot adheres firmly to BG-coated implants, indicating that BG-coated implants have higher wettability and stronger adhesion to the surrounding soft tissue than machined bare titanium implants (Mistry et al., 2016).

### 5.5 Antibacterial properties

Antibacterial properties are an essential characteristic in grafts implanted clinically, which refers to the grafts’ ability to reduce microbial growth on their surfaces. The formation of a biofilm is the first stage of bacterial growth, which also inhibits the proliferation of osteoblasts.

Better antibacterial properties are reflected in larger bacterial inhibition zones, lower reduction rates, fewer colony-forming units, and lower minimum inhibitory concentrations. A study has shown that as the content of silver ions in the coating increased, the number of bacteria on the sample surface decreased significantly and the bacterial inhibition area on the surface became larger [Figures 7B(a–f)] (Fu et al., 2017b). The cobalt- and Ti-doped glass coatings have better antibacterial properties than traditional 58S glass (Lung et al., 2021). As more zirconium oxide is added to samples, they exhibit a higher ability to inhibit bacterial growth (Bargavi et al., 2020), whereas borate-based glass exhibits worse antibacterial properties (Rau et al., 2020). Glass composited with drugs can help eradicate bacteria (Zarghami et al., 2021). By observing bacteria in saliva on the implant surface, Costa et al. (2020) found that fewer pathogenic bacteria were observed on the surface.

The antibacterial property is mainly increased by doping silver and other metallic ions in the manufacturing of chitosan composted coatings and in combination with antibiotics like tetracycline and vancomycin. The inhibition of bacteria by silver ions is mainly due to direct contact, which results in the deformation of cell membranes (Lung et al., 2021) and ROS in bacteria (Fu et al., 2017b). The electrostatic effects leading to changes in cell membrane permeability, thereby influencing cell signal transduction and production of ROS, are the main reasons for antibacterial properties caused by metallic compounds (Lung et al., 2021). Due to the slow-release behavior, BG containing chitosan and vancomycin has higher bactericidal effects (Zarghami et al., 2020). Chitosan increases cell membrane permeability, leading to the release of intracellular substances and thus causing cell death (Ordikhani et al., 2016). The composite coating of bioactive glass as a drug carrier can also significantly improve antimicrobial properties. BG has a rougher surface (Matter et al., 2021) and creates a more alkaline biological environment (Echezarreta-López and Landin, 2013; Brauer, 2015) through dissolution and ion release, where bacteria grow poorly.

In *in vivo* experiments, the plaque index and gingival recession were assessed to evaluate the degree of oral hygiene (Silness and Löe, 1964; Kassab and Cohen, 2003). A clinical trial proved that patients with BG-coated implants have reduced plaque index and gingival recession and, therefore, reduced occurrence of oral disease (Mistry et al., 2016).

## 6 Conclusion and future prospects

Bioactive glasses are widely researched because of their good biological properties; however, poor mechanical properties limit their clinical applications. Suitable coating technologies are essential for the performance of glass coatings’ relevant properties. This work systematically reviews the coating technology of BG on the surface of Ti and its alloys and summarizes the principles of the technology, relevant parameters, and their relative advantages, providing a reliable basis for coating technology selection. BG coatings exhibit excellent cell compatibility, antibacterial and anti-inflammatory properties, and higher levels of osseointegration and osteogenesis, which indicate that BG coatings on Ti and its alloys have excellent biocompatibility and bioactivity. The doping of ions and compounding with other substances significantly improve the coatings’ performances.

However, BG-coated Ti and its alloy implants face many challenges nowadays. Adding antibacterial ions, such as Ag and Co, and compositing with drugs, such as tetracycline and vancomycin, can improve the antibacterial properties of metal implants. However, the overuse of metal ions may cause cytotoxicity and limit cell metabolism, which leads to negative tissue reactions. Therefore, the balance of antibacterial properties and cytotoxicity requires further study for coating optimization. In terms of physical properties, there are significant differences in the coefficients of thermal expansion between metals and glass materials that can lead to cracking and failure of coatings. Improving the compatibility of substrates and coatings also relies on further research.

*In vitro* experiments can filter suitable implant biomaterials in the first stage, which prevents excessive euthanization of laboratory animals and simplifies the quantification of experimental results. However, cellular metabolites cause peripheral tissue reactions, and *in vitro* studies cannot test the subsequent biological effects. Animal experiments can assess the influence of coated implants on the surrounding and distant organs while controlling different pathological models and loading conditions, making them important pre-clinical research. For BG-coated implants, advanced clinical applications require more animal experiments and clinical trials.

## Data Availability

The original contributions presented in the study are included in the article/Supplementary Material; further inquiries can be directed to the corresponding authors.
